# Prevalence of kidney stones based on metabolic health and weight criteria: reports from the national health and nutrition examination survey 2007-2018 data analysis

**DOI:** 10.3389/fphys.2025.1625100

**Published:** 2025-06-23

**Authors:** Xijie Ding, Qingchen Du, Jianxing Li, Chaoyue Ji, Endi Zhang, Weiguo Hu

**Affiliations:** ^1^ Department of Urology, Qinghai University Affiliated Hospital, School of Clinical Medicine, Qinghai University, Xining, China; ^2^ Department of Urology, Beijing Tsinghua Changgung Hospital, School of Clinical Medicine, Tsinghua University, Beijing, China

**Keywords:** metabolically healthy obesity, metabolic syndrome, obesity, kidney stones, physical activity

## Abstract

**Objective:**

Using data from the NHANES collected between 2007-2018, this study aimed to investigate the relationship between the prevalence of kidney stones and metabolically healthy obesity (MHO) and measure the effect of physical activity as a modifying factor.

**Methods:**

This cross-sectional analysis included 12,498 participants aged ≥20 years who were categorized into six groups based on their metabolic status (healthy or unhealthy) and BMI (normal, overweight, and obesity). Kidney stone history was self-reported. Weighted logistic regression models, adjusted for demographic characteristics, comorbidities, and lifestyle variables, were applied to calculate odds ratios and 95% confidence intervals. Subgroup analyses were conducted based on the degree of physical activity.

**Results:**

The overall prevalence of kidney stones was 10.20%. Participants with metabolically unhealthy obesity (MUO) had the highest prevalence of kidney stones (14.5%), followed by individuals with MHO (11.1%). After full adjustment, compared to participants with MHN, the MHO and MUO groups exhibited significantly greater risks of kidney stones (MHO: OR = 2.10, 95% CI:1.47–2.98, P < 0.001; MUO: OR = 1.98, 95% CI:1.45–2.67, P < 0.001). Physical activity was associated with a decreased risk of kidney stones, particularly among individuals with MUO. Stratified analyses revealed no significant interaction effects by age, sex, or race.

**Conclusion:**

Obesity, irrespective of metabolic health status, was significantly associated with a higher prevalence of kidney stones. Higher levels of physical activity were correlated with lower risks of kidney stones, particularly in metabolically unhealthy individuals. These results underscore the importance of managing body weight and maintaining physical activity as key strategies to prevent kidney stones.

## 1 Background

Urolithiasis is a common urological condition whose prevalence has increased worldwide over the past few decades (Peerapen and Thongboonkerd, 2023). It imposes a substantial financial burden on healthcare systems. In the United States, more than $2 billion is invested annually for treating urolithiasis (Thongprayoon et al., 2020). With the concurrent increase in the incidence of metabolic abnormalities, such as diabetes and obesity, the prevalence of kidney stones has increased (Wong et al., 2016; Moftakhar et al., 2022). With the rapid increase in the number of individuals with obesity in the United States, the incidence of kidney stones is steadily increasing, and the prevalence of kidney stones disease is nearly 20% in high-risk groups (Menke et al., 2015). Therefore, elucidating the risk factors linking these conditions is crucial for the effective management of kidney stones.

Obesity is strongly associated with metabolic abnormalities (Schulze and Stefan, 2024; Alexander et al., 2022), but a distinct subgroup of obese individuals exhibits few or no metabolic abnormalities, a state known as MHO (Kramer et al., 2013). Importantly, accumulating evidence indicates that individuals with MHO exhibit a significantly lower cardiometabolic risk compared to those with MUO (Kramer et al., 2013; Eckel et al., 2018). This metabolic heterogeneity suggests that body mass index (BMI) alone is not enough to assess obesity-related health risks. Although obesity, often measured based on BMI, and associated metabolic syndrome is an established risk factor for nephrolithiasis (Lin et al., 2020; Lo et al., 2023; Ferraro et al., 2024), the effect of the MHO phenotype on the risk of nephrolithiasis remains poorly understood. Investigating MHO, rather than BMI alone, is crucial because it allows us to assess the potential effects of metabolic health status on the risk of nephrolithiasis among individuals with obesity. Carefully designed studies assessing the relationship between MHO and kidney stones can help determine the risk of nephrolithiasis among obese individuals, thereby guiding preventive interventions.

This study aimed to explore the link between MHO and nephrolithiasis by analyzing data from NHANES. Furthermore, elucidating the protective effect of physical activity on nephrolithiasis in subjects with metabolic abnormality may have significant implications for the development of specific preventive and therapeutic strategies.

## 2 Materials and methods

### 2.1 Data source and participants

The core data for this study were obtained from the NHANES database, which is managed by the U.S. Centers for Disease Control and Prevention. The NHANES employs a sophisticated probability sampling method every 2 years to collect data reflecting the U.S. population, encompassing details on demographics, socioeconomic conditions, dietary habits, and health-related factors.

### 2.2 Population

**FIGURE 1:**
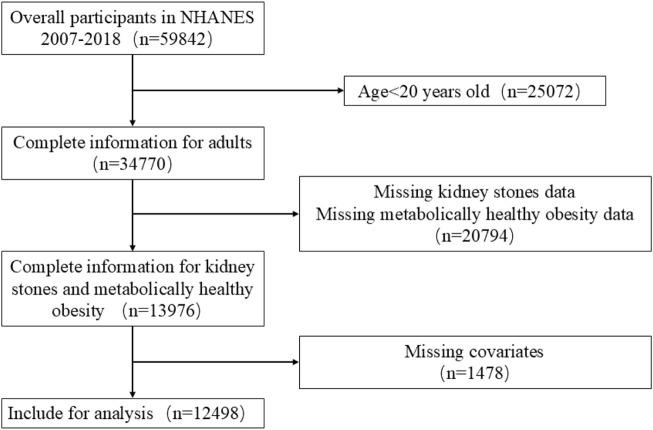
Flow diagram of participant screening. NHANES.

This study used data from six NHANES data cycles spanning 2007-2018. The inclusion criteria were as follows: (1) individuals aged 20 years or older; (2) participants providing full details on BMI, metabolic syndrome, and history of kidney stones; and (3) availability of data regarding physical activity and additional covariates. The exclusion criteria were as follows: (1) age <20 years; (2) missing data regarding the diagnosis of kidney stone or metabolic health status; (3) incomplete covariate data, e.g., age, sex, and BMI. After applying these criteria, the final sample consisted of 12,498 participants (Figure 1).

### 2.3 Exposures and outcomes

Kidney stones: The main focus of this study was to determine the incidence of kidney stones, evaluated using data from the Questionnaire (question KIQ026): “Have you ever experienced kidney stones?” Individuals who answered affirmatively were categorized as having a “kidney stone.” (Li et al., 2024).

Metabolic syndrome was defined based on the 2009 criteria (Alberti et al., 2009). Individuals meeting at least three of the following five criteria were considered to have metabolic syndrome: (1) triglyceride levels more than1.7 mmol/L or reliance on lipid-lowering medications; (2) reduced HDL-C levels, less than 1.0 mmol/L in men or 1.3 mmol/L in women, or use of cholesterol-lowering drugs; (3) elevated blood pressure (systolic ≥130 mm Hg and/or diastolic ≥85 mm Hg), or using antihypertensive drugs; (4) fasting blood glucose levels of 5.6 mmol/L or higher, a self-reported diabetes diagnosis, or use of glucose-lowering treatments; (5) elevated waist circumference (≥102 cm in men; ≥88 cm in women).

BMI was determined based on the examination data and calculated by dividing body mass in kilograms by the square of height in meters. According to World Health Organization (WHO) guidelines, participants were categorized into three groups: normal weight (18.5–24.9 kg/m^2^), overweight (25.0–29.9 kg/m^2^), and obese (>30 kg/m^2^) (Elmaleh-Sachs et al., 2023).

Using the combination of metabolic syndrome status and BMI categories, participants were divided into six groups: MHN: metabolically healthy normal weight, MHOW: metabolically healthy overweight, MHO: metabolically healthy obese, MUN: metabolically unhealthy normal weight, MUOW: metabolically unhealthy overweight, and MUO: metabolically unhealthy obese.

### 2.4 Covariates

To account for potential confounding factors, the model was adjusted for several demographic and health-related variables, including education level, sex, age, race, family income-to-poverty ratio (FIR), smoking status, coronary heart disease, gout, and physical activity. A comprehensive summary of these covariates can be found in Supplementary Table S1 (available in Supplementary Digital Content 1).

Physical activity included both moderate and vigorous activities. Moderate physical activity was defined as engagement in work-related or recreational activities lasting ≥10 min per week, accompanied by small increases in the heart rate or breathing (e.g., brisk walking, recreational volleyball, or swimming). Vigorous physical activity included activities that largely increased the heart rate or breathing (e.g., carrying heavy loads, running, competitive basketball, or construction work) for ≥10 min weekly (Li et al., 2024). Since more physical activity may reduce the risk of kidney stones (Mao et al., 2022), we conducted a logistic regression analysis, stratified by participants’ physical activity levels, to more precisely assess the association between MHO and the risk of developing kidney stones.

### 2.5 Statistical analysis

This study followed the statistical analysis protocols established by the Centers for Disease Control and Prevention (CDC), incorporating appropriate sample weights for participant selection. The baseline characteristics of the study population were outlined based on metabolic status and BMI. Continuous variables are presented as mean ± standard deviations (SD), and categorical variables are expressed as percentages (%). Survey-weighted Analysis of Variance (ANOVA) was applied to evaluate differences in continuous variables across the six metabolic-BMI groups, and survey-weighted χ^2^ tests were employed for categorical variables. Logistic regression analyses were used to assess the association between MHO and kidney stones, using both unadjusted (Model 1) and adjusted models. Model 1 was unadjusted; Model 2 was adjusted for sex, age, and race; and Model 3 was further adjusted for participant factors, including education level, FIR, smoking status, coronary heart disease, and physical activity. The magnitude of the association was determined using odds ratios (ORs) with corresponding 95% confidence intervals. Interaction effects were measured using stratified logistic regression analyses to investigate potential interactions between covariates, assess the association of MHO with kidney stones, and confirm the consistency of the findings. This approach aimed to identify the factors modifying the association between MHO and kidney stones within subgroups of physical activity. Statistical analyses were conducted using R software (developed by The R Foundation) and STATA 16.0 (produced by Stata Corporation, located in College Station, TX, USA), A two-tailed P-value <0.05 was considered statistically significant.

## 3 Results

### 3.1 Participants’ characteristics

In total, 59,842 participants were recruited for this study from 2007-2018. After applying the inclusion and exclusion criteria, 12,498 participants were included (Figure 1). A thorough and comprehensive outline of the baseline characteristics of participants is displayed in Table 1.

**TABLE 1:** Baseline characteristics of study participants by metabolic health and BMI categories, NHANES 2007-2018 (weighted).

Characteristics	Metabolically healthy			Metabolically unhealthy			
	MHN	MHOW	MHO	MUN	MUOW	MUO	P
Number(n)	3,113	2,582	1,564	394	1,619	3,226	
Age	43.7 ± 17.1	45.1 ± 15.7	42.3 ± 15.1	58.6 ± 15.5	56.7 ± 15.0	51.8 ± 15.0	P < 0.001
Sex (N/%)							
MALE	1,371 (44.0%)	1,495 (57.9%)	668 (42.7%)	150 (38.1%)	845 (52.2%)	1,561 (48.4%)	P < 0.001
FEMALE	1742 (56.0%)	1,087 (42.1%)	896 (57.3%)	244 (61.9%)	774 (47.8%)	1,665 (51.6%)	
Race(N/%)							P < 0.001
Mexican American	169 (5.4%)	253 (9.8%)	174 (11.2%)	15 (3.7%)	133 (8.2%)	300 (9.3%)	
Non-Hispanic Black	147 (4.7%)	166 (6.4%)	116 (7.2%)	14 (3.6%)	93 (5.7%)	158 (4.9%)	
Non-Hispanic White	2,172 (69.8%)	1740 (67.4%)	936 (59.9%)	277 (70.5%)	1,182 (73.0%)	2,251 (69.8%)	
Other Hispanic	258 (8.3%)	253 (9.8%)	266 (17.1%)	30 (7.5%)	105 (6.5%)	352 (10.9%)	
Other race	367 (11.8%)	170 (6.6%)	72 (4.6%)	58 (14.7%)	108 (6.7%)	165 (5.1%)	
Education level (n/%)							P < 0.001
Lower than 12th grade	392 (12.6%)	361 (14.0%)	222 (14.2%)	93 (23.6%)	288 (17.8%)	584 (18.1%)	
High school grade	616 (19.8%)	496 (19.2%)	341 (21.8%)	104 (26.5%)	455 (28.1%)	813 (25.2%)	
College grade	2,105 (67.6%)	1725 (66.8%)	1,001 (64.0%)	197 (49.9%)	876 (54.1%)	1829 (56.7%)	
Family income-to-poverty ratio (n/%)							P < 0.001
<1.3	623 (20.0%)	503 (19.5%)	357 (22.8%)	93 (23.6%)	351 (21.7%)	742 (23.0%)	
≥1.3, <3.5	1,046 (33.6%)	865 (33.5%)	638 (40.8%)	154 (39.2%)	619 (38.2%)	1,200 (37.2%)	
≥3.5	1,444 (46.4%)	1,214 (47.0%)	569 (36.4%)	147 (37.2%)	649 (40.1%)	1,284 (39.8%)	
BMI (kg/m^2^)	22.4 ± 1.7	27.2 ± 1.4	34.9 ± 4.8	23.3 ± 1.3	27.8 ± 1.4	36.5 ± 5.9	P < 0.001
HDL-C (mmol/L)	1.6 ± 0.4	1.5 ± 0.4	1.4 ± 0.3	1.3 ± 0.4	1.3 ± 0.4	1.2 ± 0.3	P < 0.001
Waist circumference (cm)	82.8 ± 7.1	95.0 ± 6.6	110.8 ± 11.8	89.9 ± 6.5	100.7 ± 6.5	117.4 ± 13.0	P < 0.001
Triglycerides (mmol/L)	1.0 ± 0.5	1.2 ± 0.7	1.1 ± 0.5	1.9 ± 1.1	2.1 ± 1.7	1.9 ± 1.6	P < 0.001
Smoking history (n/%)							P < 0.001
Smoker	1,311 (42.1%)	1,084 (42.0%)	610 (39.0%)	216 (54.8%)	869 (53.7%)	1,542 (47.8%)	
Non-smoker	1802 (57.9%)	1,498 (58.0%)	954 (61.0%)	178 (45.2%)	750 (46.3%)	1,684 (52.2%)	
Physical activity (n/%)							P < 0.001
Yes	2,497 (80.2%)	2053 (79.5%)	1,176 (75.2%)	241 (61.2%)	1,115 (68.9%)	2,132 (66.1%)	
No	616 (19.8%)	529 (20.5%)	388 (24.8%)	153 (38.8%)	504 (31.1%)	1,094 (33.9%)	
Diabetes mellitus (n/%)							P < 0.001
Yes	62 (2.0%)	88 (3.4%)	39 (2.5%)	71 (17.9%)	264 (16.3%)	742 (23.0%)	
No	3,051 (98.0%)	2,494 (96.6%)	1,525 (97.5%)	323 (82.1%)	1,355 (83.7%)	2,484 (77.0%)	
Hypertension (n/%)							P < 0.001
Yes	467 (15.0%)	524 (20.3%)	291 (18.6%)	198 (50.3%)	902 (55.7%)	1939 (60.1%)	
No	2,646 (85.0%)	2058 (79.7%)	1,273 (81.4%)	196 (49.7%)	717 (44.3%)	1,287 (39.9%)	
Coronary heart disease (n/%)							P < 0.001
Yes	84 (2.7%)	54 (2.1%)	23 (1.5%)	30 (7.6%)	91 (5.6%)	174 (5.4%)	
No	3,029 (97.3%)	2,528 (97.9%)	1,541 (98.5%)	364 (92.4%)	1,528 (94.4%)	3,052 (94.6%)	
Gout (n/%)							P < 0.001
Yes	44 (1.4%)	65 (2.5%)	38 (2.4%)	19 (4.8%)	87 (5.4%)	271 (8.4%)	
No	3,049 (98.6%)	2,517 (97.5%)	1,526 (97.6%)	375 (95.2%)	1,532 (94.6%)	2,955 (91.6%)	
Kidney stone history (n/%)							P < 0.001
Yes	196 (6.3%)	207 (8.0%)	174 (11.1%)	32 (8.1%)	199 (12.3%)	468 (14.5%)	
No	2,917 (93.7%)	2,375 (92.0%)	1,390 (88.9%)	362 (91.9%)	1,420 (87.7%)	2,758 (85.5%)	

Among the 12,498 participants, 5,239 were metabolically unhealthy, and 7,259 were metabolically healthy. Of the 7,259 metabolically healthy individuals, 3,534 (48.68%) were male, and 3,725 (51.32%) were female. Among the 5,239 metabolically unhealthy participants, 2,556 (48.79%) were male, and 2,683 (51.21%) were female. Among metabolically healthy participants, 3,113 (42.9%) had normal weight, while only 394 (7.5%) participants with normal weight were metabolically unhealthy. In addition, metabolically healthy participants had higher education levels and higher FIRs, were younger, and suffered from a lower risk of coronary heart disease and gout. They also reported higher levels of physical activity.

The overall prevalence of kidney stones was 10.20% among all participants. In each BMI category, the prevalence of kidney stones was higher among metabolically unhealthy participants compared to metabolically healthy participants: normal weight (8.1% vs. 6.3%), overweight (12.3% vs. 8.0%), and obesity (14.5% vs. 11.1%) (P < 0.001). Furthermore, in each BMI category, participation in physical activity was more evident among metabolically healthy participants compared to metabolically unhealthy participants: normal weight (80.2% vs. 61.2%), overweight (79.5% vs. 68.9%), and obesity (75.2% vs. 66.1%) (P < 0.001).

### 3.2 Logistic regression analysis of kidney stone risk

Using the MHN group as a reference, we employed a weighted logistic regression model to determine the association between MHO and kidney stones. In univariate analysis (Model 1), among physically active individuals, the risk of kidney stones was 83% higher in the MHO group compared to the MHN group (OR = 1.83, 95% CI: 1.29–2.59, P = 0.001). The MUN group exhibited a 22% higher risk than the MHN group (OR = 1.22, 95% CI: 0.64–2.33, P = 0.554), while the MUO group exhibited a 130% increased risk (OR = 2.30, 95% CI: 1.72–3.07, P < 0.001). After adjusting for covariates in Model 2 (MHO: OR = 2.04, 95% CI: 1.43–2.91, P < 0.001; MUO: OR = 2.08, 95% CI: 1.55–2.80, P < 0.001) and Model 3 (MHO: OR = 2.10, 95% CI: 1.47–2.98, P < 0.001; MUO: OR = 1.98, 95% CI: 1.45–2.67, P < 0.001), MHO and MUO were still significantly associated with the risk of kidney stones. Interestingly, among participants who did not participate in physical activities, the risk of kidney stones was increased in the MHO group (OR = 1.98, 95% CI: 1.10–3.60, P = 0.025) and the MUO group (OR = 3.10, 95% CI: 1.95–4.92, P < 0.001) compared to the MHN group. Adjustments in Model 2 (MHO: OR = 2.27, 95% CI: 1.24–4.16, P = 0.008; MUO: OR = 2.71, 95% CI: 1.69–4.34, P < 0.001) and Model 3 (MHO: OR = 2.22, 95% CI: 1.23–4.05, P = 0.007; MUO: OR = 2.88, 95% CI: 1.83–4.52, P < 0.001) did not change these significant associations (Table 2). Furthermore, the prevalence of kidney stones tends to increase with increasing body weight.

**TABLE 2:** Association between Metabolic-BMI categories and kidney stones, stratified by physical activity status (weighted).

Physical activity	Metabolically healthy			Metabolically unhealthy		
	MHN (OR, 95%Cl), P	MHOW (OR, 95%Cl), P	MHO (OR, 95%Cl), P	MUN (OR, 95%Cl), P	MUOW (OR, 95%Cl), P	MUO (OR, 95%Cl), P
Yes						
Model 1	Reference	1.31 (0.96–1.78), 0.088	1.83 (1.29–2.59), 0.001	1.22 (0.64–2.33), 0.554	2.08 (1.47–2.94), <0.001	2.30 (1.72–3.07), <0.001
Model 2	Reference	1.27 (0.93–1.74), 0.131	2.04 (1.43–2.91), <0.001	0.96 (0.50–1.86), 0.906	1.63 (1.14–2.34), 0.008	2.08 (1.55–2.80), <0.001
Model 3	Reference	1.29 (0.94–1.71), 0.129	2.10 (1.47–2.98), <0.001	0.93 (0.49–1.85), 0.875	1.61 (1.09–2.31), 0.007	1.98 (1.45–2.67), <0.001
No						
Model 1	Reference	1.29 (0.72–2.30), 0.391	1.98 (1.10–3.60), 0.025	1.51 (0.74–3.10), 0.261	2.15 (1.27–3.64), 0.005	3.10 (1.95–4.92), <0.001
Model 2	Reference	1.24 (0.70–2.20), 0.462	2.27 (1.24–4.16), 0.008	1.21 (0.58–2.53), 0.618	1.67 (0.97–2.90), 0.066	2.71 (1.69–4.34), <0.001
Model 3	Reference	1.25 (0.71–2.19), 0.452	2.22 (1.23–4.05), 0.007	1.24 (0.61–2.62), 0.558	1.72 (0.99–2.89), 0.049	2.88 (1.83–4.52), <0.001

Model 1: Unadjusted.

Model 2: Adjusted for age, sex, race.

Model 3: Additionally adjusted for education level, FIR, smoking status, coronary heart disease (Excluding physical activities).

### 3.3 Subgroup analysis

**FIGURE 2:**
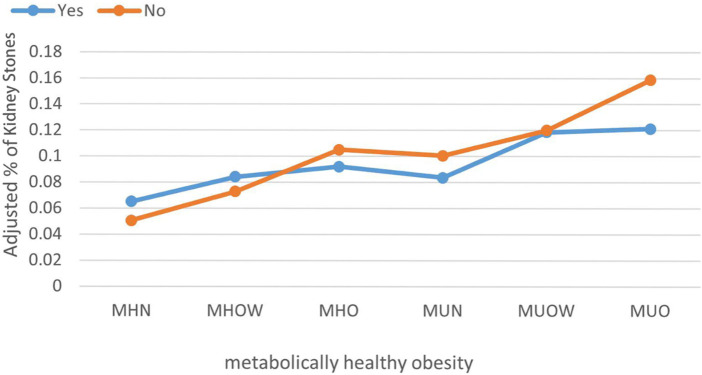
The prevalence of kidney stones among participants in different groups after adjustment. Orange indicates participants without physical activity. Blue indicates participants with physical activity. (MHN, metabolically healthy normal weight; MHOW, metabolically healthy overweight; MHO, metabolically healthy obesity; MUN, metabolically unhealthy normal weight; MUOW, metabolically unhealthy overweight; MUO, metabolically unhealthy obesity).

Additional stratified logistic regression and interaction effect analyses were conducted among participants involved in physical activities to identify variables that may modify the association between MHO and kidney stones. Formal tests for interaction revealed that age (P-interaction = 0.451), sex (P-interaction = 0.266), and race (P-interaction = 0.225) did not significantly affect the association between MHO and kidney stones. Similarly, no significant interaction effects were found for education level (P-interaction = 0.140), family income-to-poverty ratio (P-interaction = 0.416), smoking history (P-interaction = 0.661), diabetes mellitus (P-interaction = 0.202), hypertension (P-interaction = 0.940), coronary heart disease (P-interaction = 0.193), or gout (P-interaction = 0.587) (Table 3). We also assessed the association between participation in physical activity and the presence of kidney stones. Among most metabolic-BMI groups, individuals who engaged in physical activity were at a lower risk of kidney stones compared to inactive individuals (Figure 2).

**TABLE 3:** Tests for interaction: Association between Metabolic-BMI categories and kidney stones within physical activity subgroup, stratified by covariates (weighted).

Characteristics	Metabolically healthy			Metabolically unhealthy			
	MHN (OR, 95%Cl)	MHOW (OR, 95%Cl)	MHO (OR, 95%Cl)	MUN (OR, 95%Cl)	MUOW (OR, 95%Cl)	MUO (OR, 95%Cl)	P- interaction
Age (N/%)							0.451
20-39	Reference	1.62 (1.00–2.64)	1.34 (0.77–2.32)	2.51 (0.66–9.56)	2.65 (1.32–5.35)	1.77 (1.06–2.95)	
40-59	Reference	1.17 (0.68–2.02	2.81 (1.61–4.91)	0.51 (0.12–2.26)	1.76 (0.97–3.21)	2.15 (1.33–3.50)	
60-80	Reference	1.06 (0.61–1.85	1.55 (0.69–3.44)	0.97 (0.41–2.29)	1.41 (0.82–2.44)	2.01 (1.20–3.39)	
Sex (N/%)							0.266
MALE	Reference	1.16 (0.77–1.76)	1.82 (1.09–3.03)	0.48 (0.14–1.67)	1.87 (1.18–2.96)	1.98 (1.32–2.96)	
FEMALE	Reference	1.39 (0.87–2.21)	1.83 (1.14–2.92)	2.17 (0.99–4.73)	2.22 (1.31–3.76)	2.64 (1.71–3.99)	
Race(N/%)							0.225
Mexican American	Reference	1.64 (0.69–3.92)	2.35 (0.88–6.28)	1.00 (0.70–1.50)	3.77 (1.56–9.11)	2.22 (0.95–5.15)	
Non-Hispanic Black	Reference	2.66 (1.16–6.11)	1.97 (0.76–5.08)	3.78 (0.83–17.08)	1.27 (0.43–3.78)	2.92 (1.29–6.62)	
Non-Hispanic White	Reference	1.30 (0.89–1.89)	2.07 (1.34–3.19)	1.23 (0.57–2.64)	2.07 (1.37–3.11)	2.27 (1.60–3.22)	
Other Hispanic	Reference	0.92 (0.42–2.05)	1.28 (0.61–2.73)	0.64 (0.08–5.17)	2.17 (0.87–5.40)	1.64 (0.81–3.33)	
Other race	Reference	1.19 (0.46–3.10)	1.64 (0.51–5.28)	0.86 (0.18–4.18)	1.43 (0.45–4.51)	5.32 (2.34–12.08)	
Education level (n/%)							0.140
Lower than 12th grade	Reference	1.18 (0.58–2.40)	1.35 (0.54–3.35)	0.53 (0.13–2.22)	1.52 (0.71–3.27)	1.46 (0.76–2.81)	
High school grade	Reference	0.81 (0.40–1.64)	1.97 (0.96–4.06)	1.11 (0.35–3.53)	1.24 (0.58–2.66)	1.65 (0.88–3.12)	
College grade	Reference	1.51 (1.03–2.21)	1.87 (1.19–2.92)	1.54 (0.62–3.83)	2.66 (1.72–4.13)	2.80 (1.94–4.05)	
Family income-to-poverty ratio (n/%)							0.416
<1.3	Reference	1.47 (0.85–2.55)	2.25 (1.26–4.03)	1.35 (0.47–3.82)	2.12 (1.20–3.74)	2.08 (1.28–3.37)	
≥1.3, <3.5	Reference	1.09 (0.65–1.83)	1.25 (0.70–2.24)	0.81 (0.30–2.19)	1.83 (1.02–3.29)	1.78 (1.12–2.84)	
≥3.5	Reference	1.42 (0.88–2.28)	2.27 (1.28–4.01)	1.57 (0.54–4.54)	2.26 (1.33–3.85)	2.90 (1.83–4.60)	
Smoking history (n/%)							0.661
Smoker	Reference	1.17 (0.75–1.83)	1.81 (1.07–3.08)	1.11 (0.46–2.70)	1.91 (1.16–3.13)	2.23 (1.46–3.40)	
Non-smoker	Reference	1.42 (0.93–2.17)	1.86 (1.17–2.96)	1.30 (0.50–3.34)	2.21 (1.37–3.57)	2.32 (1.56–3.46)	
Diabetes mellitus (n/%)							0.202
Yes	Reference	0.93 (0.28–3.07)	6.11 (1.23–30.44)	4.13 (1.10–15.48)	2.59 (0.87–7.70)	3.79 (1.37–10.46)	
No	Reference	1.32 (0.97–1.80)	1.75 (1.23–2.50)	0.83 (0.35–2.00)	2.01 (1.38–2.92)	2.00 (1.46–2.74)	
Hypertension (n/%)							0.940
Yes	Reference	1.29 (0.71–2.32)	1.37 (0.64–2.87)	1.51 (0.65–3.50)	1.75 (1.01–3.02)	1.89 (1.15–3.12)	
No	Reference	1.26 (0.88–1.81)	1.93 (1.30–2.86)	0.39 (0.13–1.15)	1.16 (0.99–2.83)	1.82 (1.21–2.73)	
Coronary heart disease (n/%)							0.193
Yes	Reference	0.91 (0.23–3.57)	2.07 (0.24–17.97)	2.06 (0.40–10.75)	2.53 (0.73–8.83)	3.27 (1.10–9.69)	
No	Reference	1.32 (0.96–1.80)	1.83 (1.28–2.61)	1.11 (0.54–2.27)	2.05 (1.43–2.92)	2.24 (1.66–3.02)	
Gout (n/%)							0.587
Yes	Reference	0.48 (0.14–1.69)	0.49 (0.08–3.10)	0.59 (0.10–3.54)	0.33 (0.09–1.18)	0.93 (0.32–2.67)	
No	Reference	1.33 (0.97–1.83)	1.89 (1.32–2.70)	1.19 (0.60–2.38)	2.18 (1.53–3.11)	2.18 (1.61–2.94)	

## 4 Discussion

In this cross-sectional study, we explored the association between obesity and kidney stones among participants aged 20 years and older. In particular, we compared those with metabolically healthy profiles to those with metabolically unhealthy conditions. According to weighted analysis, the incidence of kidney stones was 10.20% among individuals aged 20 and older, which is close to the reported kidney stone prevalence of 9.6% in the U.S. (Mao et al., 2021; Abufaraj et al., 2021).

Our understanding of the pathogenesis of nephrolithiasis is gradually evolving, shifting from a standalone condition to one with systemic implications. Recent studies have indicated that various metabolic factors, including obesity, diabetes, and metabolic syndrome, play a crucial role in the development of kidney stones (Skolarikos et al., 2024; Singh et al., 2022). The precise mechanisms linking obesity and metabolic syndrome to nephrolithiasis remain incompletely understood, although several metabolic factors have been implicated. These factors include increased urinary excretion of uric acid and oxalate. Hyperinsulinemia can lead to hypercalciuria, and insulin resistance reduces renal ammonium production and hypocitraturia (Lo et al., 2023; Faggiano et al., 2003). Additionally, hypertension may make urinary composition conducive to stone formation, and vascular damage may impair medullary blood flow (Wang et al., 2021). Obesity is also associated with altered serum levels of calcium, phosphate, vitamin D, and uric acid, all of which may affect the risk of nephrolithiasis. Moreover, obesity upregulates systemic inflammatory markers in the blood, and inflammation is believed to increase the risk of developing kidney stones (Amin et al., 2018). In the present study, we measured the associations between metabolic abnormalities, obesity, and nephrolithiasis based on data from NHANES. Our findings reveal that irrespective of the presence of MHO or metabolically unhealthy obesity, the prevalence of kidney stones increases with BMI. Even in the presence of a normal metabolic profile, a significant independent association persists between obesity and kidney stone disease. Dietary patterns of individuals with obesity, often characterized by high caloric intake and increased consumption of animal proteins (Westbury et al., 2023), may lower urinary pH and increase uric acid excretion, thereby increasing the risk of nephrolithiasis (Thongprayoon et al., 2020). In addition, obesity may heighten the risk of nephrolithiasis by altering serum calcium concentrations (Lo et al., 2023). Physical activity serves as a protective factor against the development of kidney stones and has beneficial effects on glucose and lipid metabolism and blood pressure, thereby preserving cardiovascular function (Oja et al., 2024; Canela et al., 2023). We conducted subgroup analyses based on physical activity status. Our subgroup analysis indicated that physical activity is associated with a reduced risk of kidney stones, particularly among metabolically unhealthy individuals. The underlying mechanisms are likely multifaceted. First, physical activity can alleviate insulin resistance, thereby reducing the urinary excretion of uric acid, calcium, and phosphorus, major constituents of most stones (Weinberg et al., 2014). Second, transient dehydration caused by physical activity can enhance the release of arginine vasopressin, which promotes thirst. Higher water intake in response to thirst compensates for fluid loss, increases urine output, and dilutes stone-forming substances, thereby preventing stone formation (Mora-Rodriguez et al., 2016). Moreover, physical activity can affect the gut microbiome via multiple pathways, which in turn may reduce urinary oxalate excretion by modulating gastrointestinal oxalate metabolism, thereby preventing the formation of kidney stones (Chmiel et al., 2023). Although the associations between obesity, metabolic syndrome, and kidney stones have been established, the specific role of the MHO phenotype and the modifying effect of physical activity in these relationships did not receive sufficient attention. Our findings suggest that among metabolically unhealthy individuals, higher levels of physical activity are associated with a substantially lower risk of nephrolithiasis. These findings support recommendations for a higher level of physical activity, particularly among metabolically unhealthy individuals with low levels of activity.

To our knowledge, this is the first study to specifically investigate the association between MHO and kidney stone disease and assess the potential modifying role of physical activity. Our findings suggested that physical activity may decrease the increased risk of kidney stone formation associated with metabolically unhealthy obesity. Several limitations of this study should be acknowledged. Primarily, the cross-sectional nature of this study limited our ability to definitively establish a cause-and-effect link between MHO and kidney stone disease, highlighting the need for future longitudinal studies to validate these findings and provide deeper insights into their temporal patterns. Second, reliance on self-reported histories of kidney stones may overlook asymptomatic cases or introduce recall bias. Third, metabolic health status is dynamic and MHO may shift to MUO, but this study assessed it at a single time point, potentially misclassifying some individuals. Fourth, the lack of stratification by activity intensity (e.g., moderate vs. vigorous) or activity duration prevented dose-response analyses. Additionally, the NHANES dataset lacks information on stone composition (e.g., uric acid or calcium oxalate); therefore, we could not investigate the associations between obesity and specific stone types. Finally, despite adjustments for multiple covariates, residual confounding from factors such as dietary patterns or genetic predisposition might still affect the findings. Future studies should incorporate dynamic metabolic monitoring (e.g., continuous glucose tracking) and urinary biomarker analyses to elucidate the mechanisms linking metabolism, and obesity to nephrolithiasis, whereas clinical trials are needed to validate the efficacy of preventive strategies.

## 5 Conclusion

This cross-sectional study revealed significant associations of both MHO and unhealthy obesity with nephrolithiasis, suggesting that obesity itself may be a key factor associated with nephrolithiasis regardless of metabolic status. Notably, higher levels of physical activity were associated with lower risks of nephrolithiasis, particularly among metabolically unhealthy individuals. These findings support the role of physical activity as a potential protective factor, which may act through improved insulin sensitivity, reduced urinary excretion of stone-forming metabolites, and increased hydration. However, the cross-sectional design limits causal inference, and self-reported history of kidney stone may introduce recall bias. Future longitudinal studies with dynamic metabolic monitoring and urinary biomarkers are needed to validate these associations and elucidate the underlying pathways. Clinically, our findings recommend weight loss and physical activity to lower the risk of nephrolithiasis, especially in high-risk individuals with obesity.

## Data Availability

The original contributions presented in the study are included in the article/Supplementary Material, further inquiries can be directed to the corresponding author.

## Abbreviations

MHN, metabolically healthy normal weight; MHOW, metabolically healthy overweight; MHO, metabolically healthy obese; MUN, metabolically unhealthy normal weight; MUOW, metabolically unhealthy overweight; MUO, metabolically unhealthy obese; FIR, family income-to-poverty ratio; CDC, Centers for Disease Control and Prevention; NHANES, National Health and Nutrition Examination Survey; BMI, Body Mass Index; WHO, World Health Organization.
