# Supplementary material for: Prevalence of kidney stones based on metabolic health and weight criteria: reports from the national health and nutrition examination survey 2007-2018 data analysis
Source: Front Physiol. 2025 Jun 23;16:1625100. doi: 10.3389/fphys.2025.1625100 (PMC12230011; doi:10.3389/fphys.2025.1625100)
Supplement: Supplementary file 1 [file DataSheet1.PDF]

Supplemental Digital Content

Supplementary table 1. Detailed description and options of covariables in the NHANES database.

Covariables	Description in the NHANES database
Age	Age
Race	Race (Mexican American, Other Hispanic, Non-Hispanic Black, Non-Hispanic White, Other Race)
Education level	Education level (lower than 12th grade, high school grade, college grade)
Family income-to-poverty ratio	A ratio of family income to poverty (< 1.3, 1.3–3.5 and >3.5)
Smoking history	Participants were asked whether they smoked at least 100 cigarettes in life (yes and no)
Hypertension	Participants were diagnosed as HTN if their average blood pressure was above 140/90 mmHg. Average blood pressure was calculated by the following protocol: the diastolic reading with zero is not used to calculate the diastolic average. If all diastolic readings were zero, then the average would be zero. If only one blood pressure reading was obtained, that reading is the average. If there is more than one blood pressure reading, the first reading is always excluded from the average. Participants were asked whether they had high blood pressure (yes and no)
Coronary heart disease	Patients were asked whether they have been told they had coronary heart disease (yes and no)
Gout	Patients were asked whether they have been told they had Gout (yes and no)
Diabetes mellitus	Patients were asked whether they have been told they had Diabetes mellitus (yes and no)
